# Multi-arm Cost-Effectiveness Analysis (CEA) comparing different durations of adjuvant trastuzumab in early breast cancer, from the English NHS payer perspective

**DOI:** 10.1371/journal.pone.0172731

**Published:** 2017-03-01

**Authors:** Caroline S. Clarke, Rachael M. Hunter, Ian Shemilt, Victoria Serra-Sastre

**Affiliations:** 1 UCL Research Department of Primary Care and Population Health, University College London, London, United Kingdom; 2 UCL Institute of Education, University College London, London, United Kingdom; 3 Department of Economics, City, University of London, London, United Kingdom; University of Nebraska Medical Center, UNITED STATES

## Abstract

**Background:**

Trastuzumab improves survival in HER2+ breast cancer patients, with some evidence of adverse cardiac side effects. Current recommendations are to give adjuvant trastuzumab for one year or until recurrence, although trastuzumab treatment for only 9 or 10 weeks has shown similar survival rates to 12-month treatment. We present here a multi-arm joint analysis examining the relative cost-effectiveness of different durations of adjuvant trastuzumab.

**Methods and findings:**

Network meta-analysis (NMA) was used to examine which trials’ data to include in the cost-effectiveness analysis (CEA). A network using FinHer (9 weeks vs. zero) and BCIRG006 (12 months vs. zero) trials offered the only jointly randomisable network so these trials were used in the CEA. The 3-arm CEA compared costs and quality-adjusted life-years (QALYs) associated with zero, 9-week and 12-month adjuvant trastuzumab durations in early breast cancer, using a decision tree followed by a Markov model that extrapolated the results to a lifetime time horizon. Pairwise incremental cost-effectiveness ratios (ICERs) were also calculated for each pair of regimens and used in budget impact analysis, and the Bucher method was used to check face validity of the findings. Addition of the PHARE trial (6 months vs. 12 months) to the network, in order to create a 4-arm CEA including the 6-month regimen, was not possible as late randomisation in this trial resulted in recruitment of a different patient population as evidenced by the NMA findings. The CEA results suggest that 9 weeks’ trastuzumab is cost-saving and leads to more QALYs than 12 months’, i.e. the former dominates the latter. The cost-effectiveness acceptability frontier (CEAF) favours zero trastuzumab at willingness-to-pay levels below £2,500/QALY and treatment for 9 weeks above this threshold. The combination of the NMA and Bucher investigations suggests that the 9-week duration is as efficacious as the 12-month duration for distant-disease-free survival and overall survival, and safer in terms of fewer adverse cardiac events.

**Conclusions:**

Our CEA results suggest that 9-week trastuzumab dominates 12-month trastuzumab in cost-effectiveness terms at conventional thresholds of willingness to pay for a QALY, and the 9-week regimen is also suggested to be as clinically effective as the 12-month regimen according to the NMA and Bucher analyses. This finding agrees with the results of the E2198 head-to-head study that compared 10 weeks’ with 14 months’ trastuzumab and found no significant difference. Appropriate trial design and reporting is critical if results are to be synthesisable with existing evidence, as selection bias can lead to recruitment of a different patient population from existing trials. Our analysis was not based on head-to-head trials’ data, so the results should be viewed with caution. Short-duration trials would benefit from recruiting larger numbers of participants to reduce uncertainty in the synthesised results.

## Introduction

Breast cancer is the most common cancer in women in the UK [1] and US [2]. Around 20–30% of women diagnosed with early-stage breast cancer have tumours over-expressing the human epidermal growth factor receptor 2 (HER2) protein, and/or exhibiting HER2/neu gene amplification [3]. In women whose tumours are HER2-positive (HER2+) and therefore more aggressive, addition of trastuzumab has been found to lead to significantly increased disease-free and overall survival [4,5,6]. The budget impact of trastuzumab is high [7], mostly due to the drug’s high cost, and the most serious adverse effect observed is cardiac dysfunction [8,9]. The optimum treatment regimen for trastuzumab is still being explored, including optimal duration and timing of administration, with which chemotherapy/ies, and in what order [10,11].

In the UK, trastuzumab is licensed for the treatment of early breast cancer, to be given 3-weekly after chemotherapy for one year or until progression [7,12]. The manufacturer’s initial marketing authorisation application to NICE focused on data from the European HERA trial [13] and the North American N9831 and B31 trials of long-duration (12 months’) trastuzumab [14,15,16]. However, there is also evidence regarding the efficacy of a shorter regimen from the FinHer trial [17,18], which found that 9 weeks’ trastuzumab produced improved distant-disease-free survival (DDFS) over zero trastuzumab in the subgroup of HER2+ patients, and from the E2198 trial [19,20], which found that disease-free and overall survival (DFS and OS) were equivalent in the 10-week and 14-month arms.

Duration of trastuzumab treatment is expected to have an impact on both costs and clinical outcomes, so the aim of this paper was to examine the relative cost and effectiveness of different durations of adjuvant trastuzumab, using the results of all eight adjuvant trastuzumab in early breast cancer trials published to date that compare different adjuvant trastuzumab durations. These are: B31 [14], BCIRG006 [21], E2198 [20], FinHer [18], HERA [13], N9831 [14], PACS-04 [22], and PHARE [23], published as seven analyses (B31 and N9831 were analysed jointly by the original investigators [14]).

To determine which trials’ data could appropriately be included in the CEA model, network meta-analysis (NMA) was used, then the CEA was run as a multi-arm joint analysis using the trastuzumab durations highlighted as appropriate for inclusion by the NMA. We also used the absolute per-arm costs and outcomes from the CEA to calculate pairwise ICERs to compare the results of our multi-arm joint analysis to the results of existing pairwise analyses, in order to investigate the face validity of our work. Finally, to further corroborate our findings, the Bucher method was used to indirectly compare the survival and adverse event hazard ratios of different non-zero durations of trastuzumab, providing confidence intervals to indicate whether any differences found are statistically significant.

## Methods

### Trial identification

An initial set of eligible trials published up to February 2010 was identified in the 2012 Cochrane Review, “Trastuzumab containing regimens for early breast cancer” [10], which reported a systematic review of randomised controlled trials (RCTs) “comparing the efficacy and safety of trastuzumab alone, or in combination with chemotherapy, or no treatment, or standard chemotherapy alone, in women with HER2-positive early breast cancer, including women with locally advanced breast cancer”. Further electronic searches were performed to identify additional RCTs published up to January 2016, using keyword searches (combinations of “trastuzumab”, “Herceptin”, “(early) breast cancer” and “adjuvant”) of Web of Science Core Collection Citation Indexes (https://www.webofknowledge.com/ –all databases), the main clinical trial registries associated with the US (clinicaltrials.gov), EU (www.clinicaltrialsregister.eu), and the WHO (www.isrctn.com), and the grey literature. Search results were screened by one author, who applied the same inclusion/exclusion criteria used in the Cochrane Review to identify and select eligible RCTs, with final inclusion decisions based on full-text assessments.

### Network meta-analysis

Four different durations of trastuzumab have been investigated in clinical trials: zero, short (9 or 10 weeks), medium (6 months), and long (12 or 14 months). The aim of this analysis was to synthesise as much information as possible from the published studies to be included in the CEA model. To do this, we examined the published results and used network meta-analysis (NMA, also called mixed treatment comparison or multivariate meta-analysis) [24] to determine which trials’ data could be combined together in the CEA. The same stringent criteria governing appropriate trial inclusion in pairwise meta-analysis [25,26] also apply to network meta-analysis (NMA) [24]. For NMA results to be meaningful, the transitivity assumption states that all trials included in the network must be “jointly randomisable” [27], i.e. it would theoretically be possible for the trials to be considered part of one super-trial, where all patients who were involved could potentially have been randomised to any of the arms, implying that the exclusion/inclusion criteria and timing of randomisation are comparable across included trials. Table 1 gives information regarding these points for the seven published sets of results listed above, and some of these characteristics are also summarised in the Gantt chart in S1 Fig. Although there are eight trials, the results of trial B31 and N9831 were analysed and published jointly by the original investigators due to similarities in the trials’ design, hence there are seven sets of results.

**Table 1:** Details for all trials on arms, randomisation timing, endpoint types reported (DDFS = distant disease-free survival; DFS = disease-free survival; OS = overall survival), numbers of events, sample sizes, trial registration ID numbers and lengths of follow-up. Person-years along with the appropriate event numbers are the network meta-analysis (NMA) input data. See S1 Fig for further details on arms.

Trial dataset	Randomisation timing	Endpoint types	Arms compared	Arm brief descriptions	Node	No. proxy events	No. OS events	No. cardiac events	Sample size	Follow-up (years)	Person-years	Ref.	Clinical trial registration ID number
B31/ N9831	Randomised at start	Locoregional, contralateral, distant (metastases), new primary tumour (non-breast), death.	12 months (concomitant with taxane);	Intervention (12m con later)	2	289 (*DFS*)	146	62	2028	3.9	7909.2	[14]	NCT01424865 NCT00898898
			Zero.	Control (zero)	3	489 (*DFS*)	228	22	2017	3.9	7866.3		
HERA	Randomised after chemo, before trastuzumab	Locoregional, contralateral, distant (metastases), new primary tumour (non-breast), death.	12 months (sequential, after anthracycline and taxane);	Intervention (12m seq)	1	218 (*DFS*)	59	36	1703	2	3335	[33]	NCT00045032
			Zero.	Control (zero)	3	321 (*DFS*)	90	3	1698	2	3325.3		
PACS-04	Randomised after chemo, before trastuzumab	Locoregional, contralateral, distant (metastases), death.	12 months (sequential, after anthracycline or anthracycline/taxane);	Intervention (12m seq)	1	68 (*DFS*)	35	4	260	3.9	1018.3	[22]	NCT00054587
			Zero.	Control (zero)	3	80 (*DFS*)	28	1	268	3.9	1049.7		
E2198	Randomised at start	Locoregional, contralateral, distant (metastases), new primary tumour (non-breast), death.	“14 months”: 10 weeks, 3-month gap for anthracycline, 12 months of trastuzumab alone;	Control (14m with gap)	10	33 (*DFS*)	23	4	112	6.4	718.7	[20]	NCT00003992
			10 weeks (concomitant with taxane, before anthracycline).	Intervention (10w)	11	27 (*DFS*)	18	3	115	6.4	737.9		
BCIRG 006	Randomised at start	Distant (metastases), death	12 months (concomitant with taxane, no anthracycline, i.e. starts at randomisation);	Intervention (12m con at R)	5	214 (*DDFS*)	113	4	1075	5.4	5822.9	[21]	NCT00021255
			12 months (concomitant with taxane, after anthracycline);	Intervention (12m con later)	8	185 (*DDFS*)	94	21	1074	5.4	5817.5		
			Zero.	Control (zero)	9	257 (*DDFS*)	141	7	1073	5.4	5812.1		
FinHer	Randomised at start	Distant (metastases), death	9 weeks (concomitant with taxane, before anthracycline);	Intervention (9w)	4	4 (*DDFS*)	3	1	54	5.2	279	[18]	ISRCTN76560285
			Zero.	Control (zero)	9	15 (*DDFS*)	10	0.5	58	5.2	299.7		
PHARE	Randomised after chemo and after 6 months’ trastuzumab	Distant (metastases), death	12 months (mixed sequential and concomitant, no breakdown of chemo regimens);	Control (12m seq/con)	7	108 (*DDFS*)	66	96	1690	3.5	5985.4	[23]	NCT00381901
			6 months (mixed sequential and concomitant, no breakdown of chemo regimens).	Intervention (6m seq/con)	6	141 (*DDFS*)	93	32	1690	3.5	5985.4		

#### Categorisation of trials

We categorised trials into jointly randomisable networks, taking into consideration randomisation timing, differences in timing of trastuzumab administration, and differences in endpoints, i.e. distant-disease-free survival (DDFS) or disease-free survival (DFS) (referred to jointly as (D)DFS). The distinction between types of survival endpoints is important because DDFS includes only metastasis and death, whereas DFS additionally includes ipsilateral/local, locoregional and contralateral recurrences, as well as new non-breast tumours in all DFS-reporting studies except PACS-04. Information on these points is given in Table 1. Authors of original publications reporting only DFS were contacted to request breakdowns by recurrence type to allow DDFS event rates to be used for all trials, but no responses were received. Differences in timing of administration of trastuzumab focused on whether the administration was concomitant with or sequentially after taxane, as it is understood that concomitant administration with taxane carries significant survival benefits [28].

We have excluded some arms on the basis that certain treatments would not be offered in the UK: the vinorelbine arm of the FinHer trial was excluded as it is not recommended by NICE, and the 2-year arm of the HERA study was omitted as there is no suggestion that the length of trastuzumab duration should be increased [29]. The 12-month sequential arm of the N9831 study [30] was not included as it was omitted from the published joint analysis of 12-month concomitant trastuzumab vs. zero trastuzumab in the N9831 and B31 studies [14] because the B31 trial did not have a corresponding 12-month sequential arm. In order therefore to avoid having two separate nodes that both contained patients from the N9831 12-month concomitant arm in the network, we omitted the comparison of the N9831 12-month sequential and concomitant arms [30].

**Fig 1:**
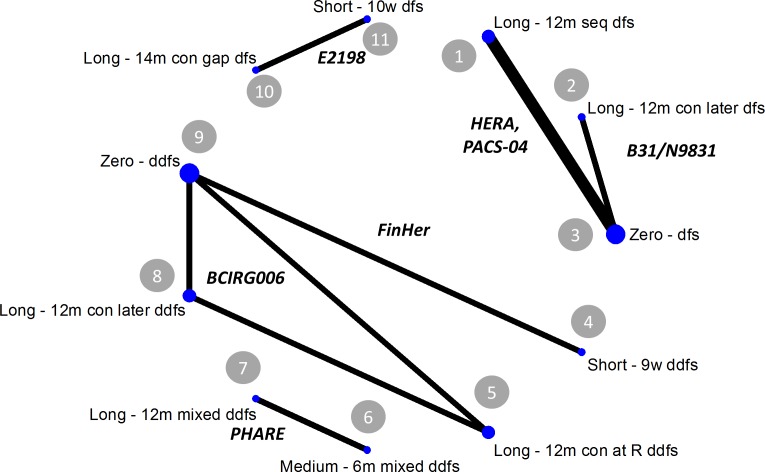
Full 11-node map, showing the four distinct jointly randomisable networks made up of the different regimens, characterised by trastuzumab duration, timing of administration, and type of disease-free endpoint reported. (Key: 14m = 14 months’ trastuzumab; 12m = 12 months’ trastuzumab; 6m = 6 months’ trastuzumab; 10 = 10 weeks’ trastuzumab; 9w = 9 weeks’ trastuzumab; gap = 3-month gap in trastuzumab administration; con = given concomitantly with taxane; seq = given sequentially with taxane; mixed = results were not broken down by administration; at R = trastuzumab begun at randomisation; later = trastuzumab begun some months after randomisation; ddfs = distant disease-free survival; dfs = disease-free survival; os = overall survival.).

When taking into consideration the differences in treatment modalities, and according to the features listed in Table 1, eleven separate treatment categories are identified, and these along with their connectivities are shown in Fig 1. The 11 categories (nodes) are split into four networks as follows: the PHARE trial forms a 2-node network by itself; the E2198 trial forms a second 2-node network; the HERA, PACS-04, and B31/N9831 trials form a 3-node network; and the BCIRG006 (three arms) and FinHer trials form a 4-node network. All lines represent a single trial connecting the two arms, except for the heavier line between nodes 2 and 4, which represents two trials making this comparison; the size of the blue dot at each node represents numbers of patients. Note that all sets of results also report OS and number of symptomatic cardiac events as well as either DFS or DDFS.

#### Ranking of treatment regimens

Decisions made regarding categorisation of trial arms into network nodes are tested by ranking the arms included in a network in order of which is “best” for a given outcome measure. This ranking is expressed as the likelihood (i.e. area under the cumulative probability curve) that a given arm in a network is the “best” for that particular outcome, i.e. reports the fewest events (deaths, onsets of metastasis, or symptomatic cardiac events). The arm with the highest probability of being best for that outcome is ranked first, and so on.

Stata 14’s mvmeta command [31] was implemented via the network package [32]. The main NMA command used was network setup d e, where *d* is the number of events and *e* the number of person-years. Input values for the trial arms are given in Table 1. The sucra command (“surface under the cumulative ranking curve”) calculates the area under the cumulative probability curve and ranks the regimens in order of efficacy according to each outcome measure.

### Cost-effectiveness model

Decision analytic modelling was used, to allow: utilities, costs, trial event data and other inputs collected from the published literature and other publicly available sources to be synthesised; costs and effects to be modelled beyond the observed timeframes of the trials; and the effects of uncertainty about the value of input parameters to be examined via sensitivity analyses. The cost-effectiveness model was written in Microsoft Excel 2007.

The model structure was determined after the NMA had been completed, as the decision regarding which trials to include in the CEA informed the choice of disease states, upon which are based all other decisions regarding state utilities, costs and transition probabilities to be used in the CEA model. Therefore we briefly outline one aspect of the NMA’s findings here to allow discussion of the CEA methodology.

The results of the NMA showed that the only jointly randomisable network with more than two trastuzumab durations was BCIRG006/FinHer (nodes 4, 5, 8, and 9 in Fig 1). We combined BCIRG006’s two 12-month nodes into a single node in the CEA despite apparent differences in timing of trastuzumab (see Table 1), since the original trial team reported that survival rates in these two arms are in fact not statistically significantly different[21] and we tested this assumption using the SUCRA command and via other sensitivity analyses described below. These trials reported DDFS and OS events, meaning that the Markov states used in the CEA were distant disease free (DDF), metastatic disease, and dead. The data included in the CEA’s three arms were therefore: (i) zero trastuzumab (FinHer’s docetaxel subgroup and BCIRG006); (ii) 9 weeks’ adjuvant trastuzumab (FinHer’s docetaxel subgroup); and (iii) 12 months’ adjuvant trastuzumab (BCIRG006’s two 12-month arms: TCH and AC-TH, see S1 Fig). UK costs and breast cancer monitoring patterns were used, adopting the English National Health Service (NHS) payer perspective.

**Fig 2:**
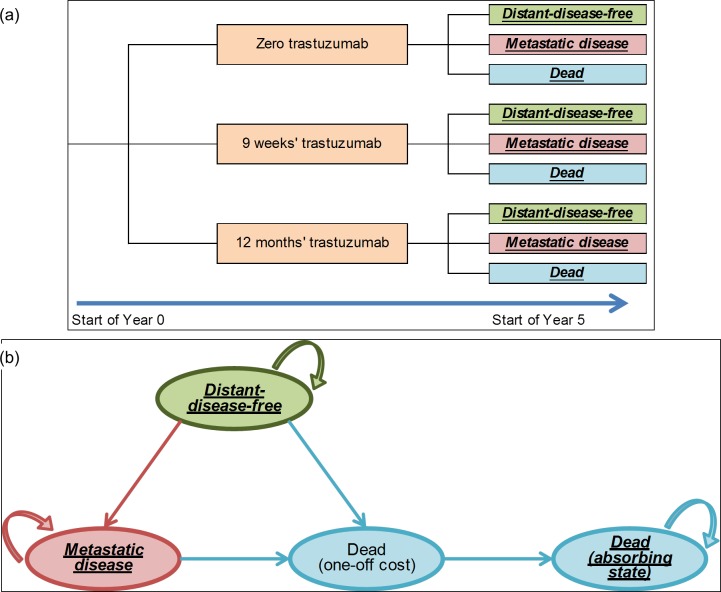
(a) Decision tree schematic showing pathway covered by the CEA’s 5-year decision tree; (b) Markov model schematic showing model structure used from year 5 onwards.

Year 0 of the CEA model began with administration of trastuzumab. Five-year survival data from FinHer [18] and BCIRG006 [21] were used to populate the decision tree and the results were extended to the lifetime horizon via a Markov model with quarterly cycles. The simulation used a cohort of 100 patients per arm of the decision tree at Year 0 (see Fig 2A). After 5 years, the patient cohort moved from the decision tree into the Markov model, with the proportion of patients in each health state in the Markov model at the beginning of year 5 determined by the proportion of patients in each arm of the decision tree at the end of year 4 (see Fig 2B).

#### Markov structure, states and transition probabilities

The Markov model included 5-year tunnel states up until 25 years from the start of trastuzumab administration, to allow for age-related changes in utility weights [34] and transition probabilities for all-cause mortality [35]. The probabilities of progression from disease-free to distant disease, and from distant disease to death, were taken from a previously published CEA based on the FinHer trial data [36] as this analysis provided appropriate Markov transition probabilities for this patient group, corresponding to the specific health state transitions in our three-state model. Costs and utilities were applied to patient cohorts at the beginning of each cycle. It was assumed that the probability of developing metastatic disease was the same regardless of trastuzumab treatment history [37], and that this risk decreased gradually to zero by year 20. Those developing metastatic disease did not return to the DDF state. The probability of death after metastasis was derived from the literature [38]. The structure of the Markov model is shown in Fig 2B and all inputs are listed in S1 Table. Patients entered the decision tree aged 52 years and the lifetime horizon used was 48 years, up to a mean age of 100 years.

#### Utilities

Utility values for the health states were derived from regression analyses published as part of a recent systematic review of utilities for breast cancer patients [39]. Upper and lower limits were derived from the highest and lowest values in the regression analysis [39]. Patient groups were sub-divided into those that suffered a symptomatic cardiac event and those that did not, and the utility weight of the former was modified during the decision tree by a multiplier of 0.60 [40,41] for a mean time period of 3 months (range 1–6 months) [37]. The utility values for the decision tree arms “metastatic disease” and “dead” were calculated assuming that the event, namely metastatic disease onset or death, depending on the arm, occurred at a point chosen to be halfway through the decision tree, i.e. 2.5 years after the start. Sensitivity bounds where the transition instead occurred at 0.5 or 4.5 years were investigated to test the validity of this decision. The utility applied to the decision tree arm “dead” assumes on average that the people who die are DDF for 1.25 years, have metastatic disease for 1.25 years, and then die at 2.5 years and these assumptions were also tested. These values and all other inputs are summarised in S1 Table.

#### Costs for monitoring, recurrence and death

Monitoring and other treatment costs were taken from 2013–14 NHS reference costs. Calculations of decision-tree arm costs used the same assumptions as those described above for the utility weights. DDF patients attended breast cancer clinic for three years or until metastasis. No costs were included for procedures or drugs common to all arms, which included chemotherapy. Cardiac monitoring patterns and costs were included according to trial protocols. In the FinHer trial, one patient who received trastuzumab, and two who did not, suffered symptomatic heart failure. It was not reported which chemotherapy agent these patients had received (docetaxel or vinorelbine), so we assumed they were split evenly between the two chemotherapy arms. Numbers of cardiac events recorded in both trials are given in Table 1. The reference cost year was 2013–14, adjusted where required using the HCHS Pay and Price Inflation Indices [42].

#### Trastuzumab costs

The dosing schedules in each arm of the model were according to the respective trial protocol. NHS reference costs for procurement and delivery of expensive chemotherapy regimens were used. The standard of care was updated in 2013 by NICE [43] to include subcutaneous (SC) administration as well as intravenous (IV), but the difference in price is small, so no corresponding sensitivity analysis has been performed (the SC regimen costs 1% more than the average cost for the IV regimen over 12 months, and 7% less over 9 weeks; British National Formulary). It is possible that there may be a reduction in the price of trastuzumab at some point in the next few years due to Herceptin’s (brand name of trastuzumab) patent expiry (Europe in 2014, USA in 2019). However, any reduction in the price would not be expected to take effect for at least 7–8 years after patent expiry, with uncertain impact on the market share of Herceptin since this depends on the production of reliable and acceptable generic versions [44].

#### Discounting

The base case annual discount rate used for both costs and outcomes was 3.5% [45], with bounds of 0% and 7%. Annual discount rates were adjusted to reflect the monthly cycles using Eq 1:

monthlyrate=((1+annualrate)112)−1. (1)

#### Sensitivity analyses

One-way deterministic sensitivity analyses were undertaken to investigate the effect that variation in each individual input parameter has on the overall results. In addition to this, joint Bayesian probabilistic sensitivity analyses (PSAs) were conducted, varying all input parameters simultaneously over 5,000 simulations, and using beta prior distributions for all proportions, rates and utilities; and gamma prior distributions for all costs. The alpha and beta parameters for the transition probabilities’ prior distributions were taken from the previously published Finnish CEA [36], and the method of moments was used to estimate alpha and beta for all other parameters based on their upper and lower limits discussed above [46]. Net monetary benefit (NMB) was calculated for a range of values of willingness to pay (WTP) for a quality-adjusted life-year (QALY), using Eq 2.

NMB=(QALYs×WTPperQALY)−Cost (2)

The 5,000 simulations were used to construct cost-effectiveness acceptability curves (CEACs), which show the probability of each arm being the most cost-effective at a given WTP. A cost-effectiveness acceptability frontier (CEAF) was generated to show the highest NMB at each WTP, indicating the ranges of WTP for which each arm was most cost-effective [47]. The CEA model and corresponding probabilistic sensitivity analyses were also run using each 12-month arm of the BCIRG006 study separately, to test whether the cost-effectiveness results were robust to the combination of the two 12-month arms into one.

### Bucher indirect comparison method

This analysis was done to corroborate the results of the CEA regarding the indirect comparison of the 9-week and 12-month regimens. The Bucher indirect comparison method used the OS hazard ratios that were modelled and published by the original trial teams: 9 weeks’ vs. zero trastuzumab [18] and 12 months’ vs. zero trastuzumab [21], with two HRs for the latter, one corresponding to each 12-month arm. The resultant calculated hazard ratios gave an indirect comparison of the relative efficacy of 12 months’ and 9 weeks’ trastuzumab in terms of OS and DDFS, with a 95% confidence interval.

### Pairwise comparisons

Besides the joint three-way comparison in the CEA described above and summarised in the CEAF, we also used the absolute values for each arm to calculate the pairwise incremental cost-effectiveness ratios (ICERs) comparing the three regimens using Eq 3, and compared these to other published ICERs for these pairs. This is an important face-validity test as work published by other groups has compared 9 weeks’ and zero trastuzumab [36, 48, 49, 50], and 12 months’ and zero trastuzumab [9,51] as pairwise comparisons only.

$$ICER=\frac{cost\;of\;new\;regimen-cost\;of\;old}{QALYs\;for\;new\;regimen-QALYs\;for\;old}$$ (3)

### Budget impact of switching

Finally, we examined the expected budget impact over one year of switching from the currently approved 12-month regimen to the 9-week regimen, which was expected to be considerable due to the high cost of trastuzumab [7]. This model used breast cancer incidence rates in England published for 2014 [1] with appropriate ineligibility reductions [7], and the calculated difference in costs per patient on using the shorter regimen instead of the longer, according to the CEA model discussed in this paper.

## Results

### Network meta-analysis

Of the 7 published sets of results, 3 reported OS, DDFS and numbers of cardiac events (BCIRG006 [21], FinHer [18] and PHARE [23]), and 4 reported OS, DFS and numbers of cardiac events (B31/N9831 [14], HERA [33], PACS-04 [22] and E2198 [20]). Note that the latest published results were used in all cases except for two: in HERA, patients were allowed to cross over from the zero arm to the 12-month arm after the first interim analysis, hence we used the 2-year follow-up results here [33] to avoid contamination present in the 4-year results [52]; and the latest publication for B31/N9831 did not report numbers of symptomatic cardiac adverse events so could not be used [15]. Of the trials that included a “long” arm (12 or 14 months), three administered trastuzumab concomitantly with taxane (BCIRG006 [21], B31/N9831 [14] and E2198 [20]), two gave it sequentially after taxane (HERA [33] and PACS-04 [22]), and one allowed both timings of administration to take place and reported only aggregated results (PHARE [23]). These and other points that distinguish between the different studies are given in Table 1, and the resultant separate networks combining comparable arms are shown above in Fig 1. A Gantt chart summarising the administration timings and other features of the arms is given in S1 Fig. Using these classifications, the only jointly randomisable network that contained more than one non-zero trastuzumab arm was the BCIRG006/FinHer network (see Fig 1); the randomisation timings and other features including choice of endpoint reported were compatible, therefore their results were used in the CEA.

The treatment arms in the BCIRG006/FinHer network were ranked in terms of the reported outcome measures (OS, DDFS and number of symptomatic adverse cardiac events) and the areas under the cumulative probability curves calculated via the SUCRA command are shown in Table 2. In terms of both DDFS and OS, the 9-week arm was preferred (95.9% and 85.9% chance of being the best regimen regarding each outcome, respectively) and, in terms of cardiac events, the zero and 9-week arms are ranked almost in joint first place (47.9% and 47.0% respective likelihoods of providing the best outcome in terms of lower numbers of cardiac events).

**Table 2:** SUCRA rankings for BCIRG006/FinHer network.

**DDFS (distant disease-free survival) events**			
Treatment duration	Area under cumulative probability curve	Probability that this treatment duration is best	Rank
Zero	0.5	0.0%	3.0
9 weeks	97.2	95.9%	1.1
12 months	52.0	4.1%	2.0
**OS (overall survival) events**			
Treatment duration	Area under cumulative probability curve	Probability that this treatment duration is best	Rank
Zero	2.2	0.0%	3.0
9 weeks	92.3	88.9%	1.2
12 months	55.5	11.1%	1.9
**Cardiac events**			
Treatment duration	Area under cumulative probability curve	Probability that this treatment duration is best	Rank
Zero	71.9	47.9%	1.6
9 weeks	55.0	47.0%	1.9
12 months	23.1	5.1%	2.5

We attempted to include the 6-month arm from PHARE in the CEA by forming a network where the 12-month arm of PHARE and the 12-month arms of BCIRG006 were combined in the same network node. However, the PHARE trial could not be considered sufficiently similar to the BCIRG006 and FinHer trials for this combination to take place due to selection bias introduced due to late randomisation. Most trials randomised patients before starting chemotherapy and some waited until chemotherapy had finished (HERA and PACS-04). The PHARE trial waited until patients had received both chemotherapy and 6 months of trastuzumab, before being randomised to either receive a further 6 months’ trastuzumab or to stop receiving it (see Table 1 and S1 Fig). As patients must have already survived to this point and been eligible for a further 6 months of trastuzumab before being randomised, we tested to see whether this led to PHARE patients being at lower risk of cardiac events than those recruited to other trials. As Table 3 shows, cardiac event results for the postulated 4-node network including PHARE, BCIRG006 and FinHer are that the 6-month arm has the highest probability of favourable cardiac outcomes (94.4%), followed by the 9-week arm (5.6%), and then the zero and 12-month arms are worst (0.0%). This did not pass face-validity checks, as it did not seem plausible that there could be a benefit in terms of fewer cardiac events when using 6 months’ trastuzumab, compared to using it for either 9 weeks or 12 months. If PHARE was omitted, however, there was no discernible difference between zero and short duration of treatment for the probability of cardiac events (47.9% and 47.0%, respectively). Therefore, PHARE participants cannot be regarded as having been recruited from the same patient population as those in BCIRG006 and FinHer, given the lower risk of cardiac events in the PHARE study, and PHARE cannot be included in the CEA.

**Table 3:** SUCRA rankings for network including BCIRG006, FinHer and PHARE.

**DDFS (distant disease-free survival) events**			
Treatment duration	Area under cumulative probability curve	Probability that this treatment duration is best	Rank
Zero	18.6	0.0%	3.4
9 weeks	97.5	95.5%	1.1
6 months	16.3	0.2%	3.5
12 months	67.5	4.4%	2.0
**OS (overall survival) events**			
Treatment duration	Area under cumulative probability curve	Probability that this treatment duration is best	Rank
Zero	20.8	0.0%	3.4
9 weeks	93.1	88.5%	1.2
6 months	16.3	0.3%	3.5
12 months	69.9	11.1%	1.9
**Cardiac events**			
Treatment duration	Area under cumulative probability curve	Probability that this treatment duration is best	Rank
Zero	16.8	0.0%	3.5
9 weeks	24.4	5.6%	3.3
6 months	98.1	94.4%	1.1
12 months	60.7	0.0%	2.2

### Cost-effectiveness model

The per-arm probabilistic results for the BCIRG006/FinHer cost-effectiveness model are given in Table 4. At a willingness to pay (WTP) £30,000 per QALY gained, i.e. the upper end of the generally accepted NICE threshold for cost per QALY gained (£20,000 to £30,000/QALY), 9 weeks has the highest NMB (£276,600 per patient over a lifetime), followed by zero trastuzumab, then 12 months. The zero arm had the lowest cost and resulted in fewest QALYs. The 95% confidence intervals for the numbers of QALYs in each arm calculated from the simulation results all overlap (see Table 4), indicating there is no difference in QALYs among the arms using Monte Carlo error. When considering the costs per arm, the 95% confidence intervals (CIs) for the costs of the zero and 9-week arms do overlap with each other, suggesting there is no difference, but neither overlap with the 95% CIs of the 12-month arm, suggesting that there is a difference in costs on using the 12-month regimen using Monte Carlo error.

**Table 4:** Lifetime horizon probabilistic (n = 5,000) net monetary benefit per patient (WTP = £30,000/QALY), total cost and total QALYs per patient per arm (mean and 95% CI of simulation results).

Arm	NMB per patient (WTP = £30,000/QALY)	Total cost per patient	Total QALYs per patient
Zero	£238,882	£20,552	8.6
	(£165,614 to £312,151)	(£15,269 to £25,835)	(6.4 to 10.9)
9 weeks	£276,600	£23,662	10.0
	(£190,568 to £362,632)	(£19,443 to £27,881)	(7.3 to 12.7)
12 months	£228,185	£46,859	9.2
	(£147,285 to £309,085)	(£39,311 to £54,406)	(6.7 to 11.6)

WTP = willingness to pay; QALY = quality-adjusted life-years; CI = confidence interval; NMB = net monetary benefit

### Pairwise comparisons

In the pairwise comparison of 9 weeks’ vs. 12 months’ trastuzumab, the shorter regimen dominates the longer, since it costs less and results in more QALYs. The 9-week regimen results in 0.8 more QALYs per patient than the 12-month, with a cost saving of £23,197 per patient. Pairwise comparisons of each duration to zero trastuzumab gave ICERs of £50,559 per QALY gained from using 12-month trastuzumab instead of zero, and £2,286 per QALY gained from using 9-week trastuzumab instead of zero. Any differences in the pairwise ICERs discussed here compared to those obtained using the values given in Table 4 are due to rounding.

### Budget impact of switching

In 2014 in England, 46,085 new breast cancer cases in women were reported [1], of which 70% are likely to respond to adjuvant treatment, and 22% of these will have HER2+ tumours [7], meaning that an additional 7,097 women per year could benefit from being given trastuzumab. According to current exclusion criteria, approximately 20% of these women would be ineligible for trastuzumab use, leaving 5,678 patients. Considering the impact on costs and effects of switching from the current 12-month regimen to the shorter 9-week regimen implies cost savings and a gain in QALYs, i.e. the short regimen dominates the long. The cost saving would be £132 million for this cohort of patients in England at 2014 prices and incidence rates, with a corresponding gain of 4,773 QALYs.

### Bucher indirect comparison method

Variation in OS and DDFS were found to be the second strongest drivers of differences in QALYs in the model, after variation in the Markov utility scores. Given the strength of the cost-effectiveness findings for 9 weeks compared to 12 months, we used Bucher analysis [53] to determine whether there were real significant differences in OS and DDFS between the 12-month and 9-week regimens, and test the hypothesis that the difference in QALYs is an artefact in the construction of the model, rather than the result of a true difference. The hazard ratio of 9 weeks vs. 12 months was calculated indirectly under two possible scenarios for each endpoint, one for each of the 12-month vs. zero hazard ratios reported for two 12-month arms in BCIRG006. The HRs for OS and DDFS are reported in Table 5 and are all smaller than 1, favouring 9 weeks, but not significantly smaller, suggesting an important level of uncertainty in the results, potentially due to the small patient numbers in the FinHer trial. The E2198 study, published in 2015, compared 10 weeks’ and 14 months’ trastuzumab treatment, and also found no significant difference between the short and long regimens [20], potentially also due to small patient numbers.

**Table 5:** Hazard ratios for indirect pairwise comparison of 12 months vs. 9 weeks, via the Bucher method.

	FinHer, BCIRG006 (TCH)	FinHer, BCIRG006 (AC-TH)
OS, 9w vs. 12m; HR (95%CI)	0.55 (0.17 to 1.79)	0.67 (0.20 to 2.20)
DDFS, 9w vs. 12m; HR (95%CI)	0.43 (0.15 to 1.21)	0.50 (0.18 to 1.41)

TCH = BCIRG006 arm comprising docetaxel, carboplatin and 12 months’ trastuzumab; AC-TH = BCIRG006 arm comprising doxorubicin and cyclophosphamide followed by docetaxel and 12 months’ trastuzumab; OS = overall survival; DDFS = distant disease-free survival; 9w = 9 weeks; 12m = 12 months; HR = hazard ratio; CI = confidence interval.

### Sensitivity analysis

Assumptions regarding input parameters were tested using deterministic one-way sensitivity analysis. Those inputs whose variation led to instability in the results were: variation in the Markov utility scores, potentially due to the large ranges available for these utility scores (see S1 Table); and variation in the proportions of patients in each state at the end of the decision tree, i.e. the OS and DDFS associated with each regimen. The relative sizes of each arm’s NMB were stable to variation in all other input parameters and assumptions. Additional sensitivity analysis was conducted to test the stability of the model when treating as separate each 12-month arm from the BCIRG006 trial. The means and 95% CIs for the separate 12-month arms (see S2 Table) are very similar to those in Table 4 for the combined 12-month arm, and considerations of the Monte Carlo error when comparing these to the zero and 9-week results in Table 4 lead to the same conclusions as for the combined 12-month arm. Different values for the OS and DDFS for each arm were tested to see how these changes affected the NMB. For the NMB of the 12-month arm to be as high as that of the 9-week (£276,600 per patient over the lifetime horizon, assuming no change in the 9-week survival rates), the 12-month survival rates must be such that the proportions of patients at the beginning of year 5 fall into the disease categories with the following split: 99% DDF, 1% metastatic, and zero dead. Similarly, if the 9-week patient proportions at year 5 are the same as in the 12-month arm (i.e. 81.4% distant-disease-free, 8.9% metastatic, 9.6% dead), then the NMB for the 9-week arm is £247,588 per patient, i.e. it is still preferred over both the zero and the 12-month arms (compare to NMB values in Table 4).

**Fig 3:**
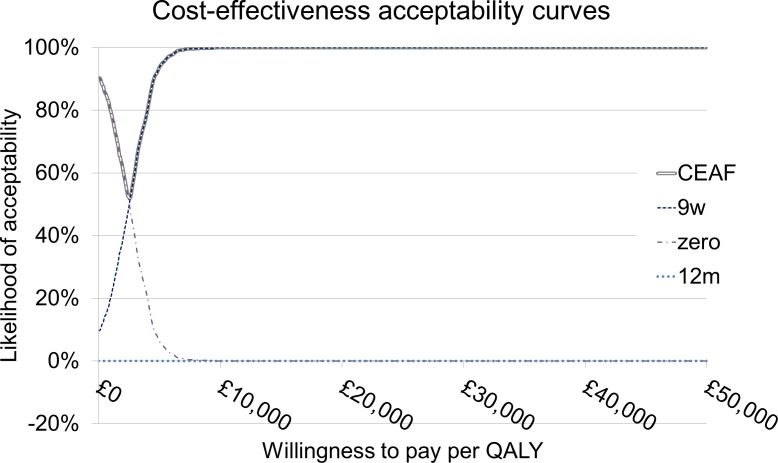
Cost-effectiveness acceptability curves for the three arms showing which duration is more cost-effective at different levels of WTP. The heavy tram line indicates the frontier (CEAF).

Probabilistic sensitivity analysis was performed and the results are shown in CEACs and the CEAF, presented in Fig 3. The zero arm has the highest probability of being cost-effective for a WTP less than £2,500 per QALY gained. For all WTP levels above £2,500/QALY, tested up to £150,000/QALY, 9 weeks has the highest probability of being cost-effective. The CEACs are calculated using the points of the absolute costs and effects plane, which is given in S3 Fig. This plot shows the spread of possible values for each arm when considering the joint uncertainties among all inputs, and shows that this uncertainty leads to considerable overlap in numbers of QALYs gained between all arms, and a clear distinction in costs between the zero/9-week arms and the 12-month arm, in agreement with the lack of overlap of the 95% confidence intervals for the NMBs in Table 4. The three red dots indicate the deterministic values for the absolute QALYs and absolute costs for each arm. Note that as there are three arms and not two, because S3 Fig does not show the incremental costs and effects; instead it shows absolute values.

## Discussion

This study considered the relative cost-effectiveness of 9-week and 12-month trastuzumab treatment regimens via multi-arm joint analysis, using NMA to aid in selection of studies for inclusion in the CEA to ensure that the joint analysis was valid. We tested the face validity of our CEA results via the Bucher method. Our results suggest that the 9-week regimen dominated the 12-month regimen as it resulted in a lower cost per patient and more QALYs. The key driver of the difference was the considerably lower cost of the shorter regimen, as the differences in QALYs gained in each arm were not significant. The deterministic sensitivity analysis suggested that an unrealistically low death rate would be required of the 12-month arm for it to show greater cost-effectiveness than the 9-week arm. There was however significant uncertainty associated with the HR for DDFS and OS for 9-week versus 12-month trastuzumab, a key driver of the model, so caution should be taken with this interpretation until additional research is conducted on the relative effectiveness of 9-week trastuzumab.

NMA can be a powerful tool for synthesising data from multiple trials. This study is, to our knowledge, the first to use this technique to investigate the relative effectiveness of different trastuzumab durations for determining inputs for a model. We note that a previous pairwise meta-analysis by Yin et al. [6] reported that using some trastuzumab (grouping 9 weeks’ and 12 months’ together) gave better survival than using no trastuzumab, and the current study goes further by considering different durations of trastuzumab separately. As with all data synthesis, the decision over what data to include is critical. What we were unable to test as part of this model was the impact of a middle ground for treatment regimen, for example 6 months. The results of the NMA show that inclusion of the only trial to test a 6-month regimen, PHARE, leads to a significant change in the ranking of treatments according to their impact on adverse cardiac events when combined with other trial data. It is likely that this is due to the study design leading to bias in selection of lower-risk patients, particularly in relation to cardiac events.

Pairwise comparisons of 9 weeks vs. zero and 12 months vs. zero give ICERs that agree with those of other CEAs examining these regimens in different countries which also found that the 9-week adjuvant trastuzumab with docetaxel followed by anthracycline-based treatment dominates or is acceptably cost-effective compared to the same regimen without trastuzumab [36,48,49,50]. Regarding the comparison of 12 months’ vs. zero trastuzumab, Kurian et al. [9] considered the B31/N9831 trials and found an ICER of US$39,982/QALY (which corresponds to £32,487 at 2013 prices) using the US perspective. The final appraisal determination by the ERG [51] for NICE used the HERA trial and found an ICER of £33,000 per QALY gained for 12 months’ vs. zero trastuzumab. Slight variations between ICERs are likely to reflect differences regarding which trials’ data were used, model structure and inputs, and other modelling assumptions made. Specifically, the model used by the manufacturer in the ERG report [54] was different from the model used here in terms of its structure (the manufacturer’s model incorporated tunnelling states for cardiac and locoregional recurrence) and input data (the manufacturer’s model mostly used 2-year data from the HERA study database). The costs, utility values and some transition probabilities used in the manufacturer’s model have not been made public, so we are unable to offer any further explanation for the difference between our ICER and theirs.

Given the obvious additional cost of longer treatment durations, and with limited evidence of benefit, more evidence on treatment durations is needed. The currently ongoing SOLD [55] and Short-HER [56] trials will provide head-to-head data on the effect of different treatment durations and will provide additional evidence on the impact of different trastuzumab administration durations in terms of OS and (D)DFS. SOLD (Synergism Or Long Duration study) is a Phase 3 randomised controlled trial (RCT) that has enrolled 2,168 patients in Finland and compares 9 weeks’ adjuvant trastuzumab (either weekly or 3-weekly) given concomitantly with docetaxel (i.e. the same as the docetaxel subgroup in the short arm of the FinHer trial), to the same regimen followed by trastuzumab monotherapy up to 1 year. The Short-HER trial is a Phase 3 non-inferiority trial to test whether the 12-month duration can be reduced to 3 months without loss of efficacy. It is possible that the benefit derived from short treatment regimens is partly due to early administration of trastuzumab concomitantly with taxane before any other chemotherapy is given[28]. If this is the case, this advantage might be lost in the 3-month arm of the Short-HER trial. Further cost-effectiveness analysis should be undertaken in the future using the results of these two trials, focusing on the question of optimal duration of trastuzumab and its impact on costs and QALYs.

Limitations of this CEA include the large uncertainties in utility values and survival rates, which lead to significant variation in the numbers of QALYs available to each arm, and therefore variations in the NMBs for each arm. Also, only three disease states are used, implying that locoregional recurrence has similar costs and utilities to those of the disease-free state. It should also be noted that the short-duration trials were not designed to be confirmatory trials regarding trastuzumab duration, hence their small patient numbers: the FinHer trial has 112 patients in the trastuzumab arm (54 of which are included in our CEA due to our exclusion of the vinorelbine arm) and the E2198 feasibility trial has 115 patients in the 10-week arm and 112 in the 14-month arm (see Table 1). As a result, limited conclusions can be drawn about the strength of their findings. No subgroup analyses (e.g. by hormone status) have been attempted, since published trial data are not reported at a sufficiently disaggregated level to enable this.

## Conclusions

This is, to our knowledge, the first multi-arm analysis that compares 9 weeks’ and 12 months’ adjuvant trastuzumab treatment (although previous studies have investigated each regimen separately in comparison with zero trastuzumab [48,50]). It is also the first network meta-analysis comparing more than two different non-zero durations of trastuzumab. This study adds to a growing body of evidence that addresses the important issue of optimal trastuzumab treatment regimens in early breast cancer [11]. It supports further investigation of the hypothesis that shorter regimens may cost less and may potentially result in equivalent, if not better, clinical outcomes than the currently approved 12-month duration. The NMA, Bucher indirect comparison, and previously published E2198 head-to-head comparison [20] all show that a short 9- or 10-week regimen could potentially be as effective as a 12- or 14-month regimen in purely clinical terms; DDFS, DFS and OS all seem to be the same under the 9-week regimen as they are for the one-year regimen, with evidence of reduced cardiotoxicity. There is still significant uncertainty in the results though meaning that further research is warranted.

We found that the 9-week regimen dominated the 12-month regimen in cost-effectiveness terms, but this is based on very little evidence because we had to drop six out of eight studies’ results as their data had not been collected and/or presented in a way that allowed comparison. Specifically, we could not include results from the only other short-duration trial, E2198, in our CEA model due to differences in endpoint reporting, and we could not include a 6-month arm due to PHARE’s late randomisation point. All trial study teams have a responsibility to their patients and to the wider public to design their trials such that the results are as comparable as possible to other work, and we suggest that these factors should be taken into close consideration when designing and reporting studies.

Future studies with short and medium treatment durations and more comparable trial designs and reporting could be incorporated into our cost-effectiveness model at a later stage, to allow for the inclusion of trastuzumab regimens of short and medium duration among compared treatment strategies.

## Supporting information

S1 Fig Gantt chart showing various design and reporting aspects of all arms from all 8 trials of adjuvant trastuzumab in early breast cancer. (DOCX)

S2 Fig Probabilities and cumulative probabilities for each treatment type. The left-hand column of plots gives the probability of each regimen being ranked in each position, i.e. first, second or third in terms of each event type. The right-hand column shows the cumulative probabilities. The area under the curve (AUC) for the cumulative probability plots is equal to a maximum value of 1 if the regimen is unequivocally the best for that event type, and 0 if it is the worst.
(DOCX)

S3 Fig Plane showing the PSA results for the absolute costs and QALYs for each of the three arms. The three red dots indicate the mean costs and QALYs for each arm.
(DOCX)

S1 Table Inputs for all parameters, showing the base case, lower and upper bounds, and the type of prior distribution used in the PSA. (DOCX)

S2 Table Lifetime horizon probabilistic (n = 5,000) net monetary benefit (WTP = £30,000/QALY), total cost and total QALYs per patient per arm (mean and 95% CI) for the two individual 12-month arms of the BCIRG006 study. (DOCX)
