# Supplementary material for: Multi-arm Cost-Effectiveness Analysis (CEA) comparing different durations of adjuvant trastuzumab in early breast cancer, from the English NHS payer perspective
Source: PLoS One. 2017 Mar 1;12(3):e0172731. doi: 10.1371/journal.pone.0172731 (PMC5383006; doi:10.1371/journal.pone.0172731)
Supplement: S1 Fig — (DOCX) [file pone.0172731.s001.docx]

**Supporting Information**

Figure S1. Gantt chart showing various design and reporting aspects of all arms from all 8 trials of adjuvant trastuzumab in early breast cancer
