# Supplementary material for: Multi-arm Cost-Effectiveness Analysis (CEA) comparing different durations of adjuvant trastuzumab in early breast cancer, from the English NHS payer perspective
Source: PLoS One. 2017 Mar 1;12(3):e0172731. doi: 10.1371/journal.pone.0172731 (PMC5383006; doi:10.1371/journal.pone.0172731)
Supplement: S2 Fig — The left-hand column of plots gives the probability of each regimen being ranked in each position, i.e. first, second or third in terms of each event type. The right-hand column shows the cumulative probabilities. The area under the curve (AUC) for the cumulative probability plots is equal to a maximum value of 1 if the regimen is unequivocally the best for that event type, and 0 if it is the worst. (DOCX) [file pone.0172731.s002.docx]

**Supporting Information**

Figure S2. Probabilities and cumulative probabilities for each treatment type. The left-hand column of plots gives the probability of each regimen being ranked in each position, i.e. first, second or third in terms of each event type. The right-hand column shows the cumulative probabilities. The area under the curve (AUC) for the cumulative probability plots is equal to a maximum value of 1 if the regimen is unequivocally the best for that event type, and 0 if it is the worst.
