# Supplementary material for: Multi-arm Cost-Effectiveness Analysis (CEA) comparing different durations of adjuvant trastuzumab in early breast cancer, from the English NHS payer perspective
Source: PLoS One. 2017 Mar 1;12(3):e0172731. doi: 10.1371/journal.pone.0172731 (PMC5383006; doi:10.1371/journal.pone.0172731)
Supplement: S3 Fig — The three red dots indicate the mean costs and QALYs for each arm. (DOCX) [file pone.0172731.s003.docx]

**Supporting Information**

Figure S3. Plane showing the PSA results for the absolute costs and QALYs for each of the three arms. The three red dots indicate the mean costs and QALYs for each arm.
