# Supplementary material for: Multi-arm Cost-Effectiveness Analysis (CEA) comparing different durations of adjuvant trastuzumab in early breast cancer, from the English NHS payer perspective
Source: PLoS One. 2017 Mar 1;12(3):e0172731. doi: 10.1371/journal.pone.0172731 (PMC5383006; doi:10.1371/journal.pone.0172731)
Supplement: S1 Table — (DOCX) [file pone.0172731.s004.docx]

**Supporting Information**

Table S1. Inputs for all parameters, showing the base case, lower and upper bounds, and the type of prior distribution used in the PSA.

| **Total cost per patient over initial five years in decision tree** | **Mean** | **Lower** | **Upper** |  | **Source** |
| --- | --- | --- | --- | --- | --- |
| 12m trastuzumab procurement and delivery cost (tree) | £27,871 | £21,294 | £34,448 | GAMMA | NHS reference costs |
| 12m trastuzumab cardiac monitoring cost (tree) | £588 | £361 | £814 |  |  |
| 9w trastuzumab procurement and delivery cost (tree) | £7,499 | £5,897 | £9,101 |  |  |
| 9w trastuzumab cardiac monitoring cost (tree) | £336 | £165 | £507 |  |  |
| Zero trastuzumab procurement and delivery cost (tree) | £0 | £0 | £0 |  |  |
| Zero trastuzumab cardiac monitoring cost (tree) | £0 | £0 | £0 |  |  |
| 12m cancer monitoring (tree), distant-disease-free at year 5 | £1,557 | £1,401 | £1,713 |  |  |
| 12m cancer monitoring and treatment (tree), metastatic at year 5 | £28,490 | £7,099 | £39,056 |  |  |
| 12m cancer monitoring and treatment (tree), dead at year 5 | £18,160 | £4,175 | £43,231 |  |  |
| 9w cancer monitoring (tree), distant-disease-free at year 5 | £1,557 | £1,401 | £1,713 |  |  |
| 9w cancer monitoring and treatment (tree), metastatic at year 5 | £28,923 | £7,099 | £48,293 |  |  |
| 9w cancer monitoring and treatment (tree), dead at year 5 | £18,593 | £4,175 | £52,468 |  |  |
| Zero cancer monitoring (tree), distant-disease-free at year 5 | £1,557 | £1,401 | £1,713 |  |  |
| Zero cancer monitoring and treatment (tree), metastatic at year 5 | £29,009 | £7,099 | £50,141 |  |  |
| Zero cancer monitoring and treatment (tree), dead at year 5 | £18,679 | £4,175 | £54,315 |  |  |
| Cardiac treatment costs if required (tree) | £636 | £212 | £1,272 |  |  |
|  |  |  | | | |
| **Percentage pts in each disease state after initial 5 yrs, 12m arm** | **Mean** | **Lower** | **Upper** |  | [21] |
| % distant-disease-free after initial 5 yrs, 12m | 81.4% | 73.3% | 89.6% | BETA |  |
| % metastatic after initial 5 yrs, 12m | 8.9% | 8.0% | 9.8% |  |  |
| % dead after initial 5 yrs, 12m | 9.6% | 8.7% | 10.6% |  |  |
|  |  |  |  |  |  |
| **Percentage pts in each disease state after initial 5 yrs, 9w arm** | **Mean** | **Lower** | **Upper** |  | [18] |
| % distant-disease-free after initial 5 yrs, 9w | 92.6% | 83.3% | 100.0% | BETA |  |
| % metastatic after initial 5 yrs, 9w | 1.9% | 1.7% | 2.0% |  |  |
| % dead after initial 5 yrs, 9w | 5.6% | 5.0% | 6.1% |  |  |
|  |  |  |  |  |  |
| **Percentage pts in each disease state after initial 5 yrs, zero arm** | **Mean** | **Lower** | **Upper** |  | [18,21] |
| % distant-disease-free after initial 5 yrs, zero | 76.0% | 73.5% | 78.4% | BETA |  |
| % metastatic after initial 5 yrs, zero | 10.7% | 9.6% | 11.8% |  |  |
| % dead after initial 5 yrs, zero | 13.4% | 12.0% | 14.7% |  |  |
|  |  |  |  |  |  |
| **Average utility during initial 5 years of decision tree** | **Mean** | **Lower** | **Upper** |  |  |
| Distant-disease-free at year 5, no cardiac event | 0.617 | 0.427 | 0.623 | BETA | [39,40] |
| Metastatic at year 5, no cardiac event | 0.567 | 0.296 | 0.650 |  |  |
| Dead at year 5, no cardiac event | 0.283 | 0.000 | 0.650 |  |  |
| Distant-disease-free at year 5, yes cardiac event | 0.605 | 0.409 | 0.619 |  |  |
| Metastatic at year 5, yes cardiac event | 0.555 | 0.283 | 0.646 |  |  |
| Dead at year 5, yes cardiac event | 0.278 | 0.000 | 0.646 |  |  |
|  |  |  |  |  |  |
| **Markov state costs (monthly)** | **Mean** | **Lower** | **Upper** |  |  |
| Distant-disease-free | £0 | £0 | £0 | GAMMA | NHS reference costs |
| Metastatic | £924 | £831 | £1,016 |  |  |
| Death (one-off cost) | £4,175 | £3,757 | £4,592 |  |  |
| Death | £0 | £0 | £0 |  |  |
|  |  |  |  |  |  |
| **Markov state utilities** | **Mean** | **Lower** | **Upper** |  |  |
| Distant-disease-free, years 5-10 | 0.617 | 0.427 | 0.623 | BETA | [34,39] |
| Metastatic disease, years 5-10 | 0.516 | 0.281 | 0.894 |  |  |
| Distant-disease-free, years 10-15 | 0.617 | 0.427 | 0.623 |  |  |
| Metastatic disease, years 10-15 | 0.516 | 0.281 | 0.894 |  |  |
| Distant-disease-free, years 15-20 | 0.614 | 0.424 | 0.620 |  |  |
| Metastatic disease, years 15-20 | 0.513 | 0.278 | 0.891 |  |  |
| Distant-disease-free, years 20-25 | 0.586 | 0.396 | 0.592 |  |  |
| Metastatic disease, years 20-25 | 0.485 | 0.250 | 0.863 |  |  |
| Distant-disease-free, years 25+ | 0.532 | 0.342 | 0.538 |  |  |
| Metastatic disease, years 25+ | 0.431 | 0.196 | 0.809 |  |  |
| Dead (one-off cost) | 0.000 | 0.000 | 0.000 |  |  |
| Dead | 0.000 | 0.000 | 0.000 |  |  |
|  |  |  |  |  |  |
| **Transition probabilities (monthly)** | **Mean** |  |  |  |  |
| Distant-disease-free > Metastatic 5-10 years | 0.380% | α=0.441 | β=115.56 | BETA | [35,36,38] |
| Distant-disease-free > Metastatic 10-15 years | 0.300% | α=0.348 | β=115.65 |  |  |
| Distant-disease-free > Metastatic 15-20 years | 0.270% | α=0.313 | β=115.69 |  |  |
| Distant-disease-free > Metastatic 20-25 years | 0.000% | 0.000% | 0.000% |  |  |
| Distant-disease-free > Metastatic 25+ years | 0.000% | 0.000% | 0.000% |  |  |
| All-cause death, aged 59 (5-10y) | 0.039% | 0.035% | 0.043% |  |  |
| All-cause death, aged 64 (10-15y) | 0.060% | 0.054% | 0.066% |  |  |
| All-cause death, aged 69 (15-20y) | 0.095% | 0.086% | 0.105% |  |  |
| All-cause death, aged 74 (20-25y) | 0.166% | 0.149% | 0.182% |  |  |
| All-cause death, aged 79 (25+y) | 0.295% | 0.265% | 0.324% |  |  |
| Death from metastatic breast cancer | 2.622% | 2.360% | 2.884% |  |  |
|  |  |  |  |  |  |
| **Discount rates (monthly)** | **Mean** | **Lower** | **Upper** | BETA |  |
| Cost discount rate | 0.29% | 0.00% | 0.57% |  | [45] |
| Outcomes discount rate | 0.29% | 0.00% | 0.57% |  |  |
