# Supplementary material for: Multi-arm Cost-Effectiveness Analysis (CEA) comparing different durations of adjuvant trastuzumab in early breast cancer, from the English NHS payer perspective
Source: PLoS One. 2017 Mar 1;12(3):e0172731. doi: 10.1371/journal.pone.0172731 (PMC5383006; doi:10.1371/journal.pone.0172731)
Supplement: S2 Table — (DOCX) [file pone.0172731.s005.docx]

**Supporting Information**

Table S2. Lifetime horizon probabilistic (n=5,000) net monetary benefit (WTP=£30,000/QALY), total cost and total QALYs per patient per arm (mean and 95% CI) for the two individual 12-month arms of the BCIRG006 study.

| **Arm** | **NMB (WTP = £30,000/QALY)** | **Total cost per patient** | **Total QALYs per patient** |
| --- | --- | --- | --- |
| 12 months (TCH only) | £228,495 | £47,119 | 9.1 |
|  | (£144,721 to £304,268) | (£39,335 to £54,904) | (6.7 to 11.5) |
| 12 months (AC-TH only) | £233,097 | £46,676 | 9.3 |
|  | (£150,093 to £316,101) | (£38,860 to £54,492) | (6.8 to 11.8) |
